# Supplementary material for: Sex differences in associated factors for age-related hearing loss
Source: PLoS One. 2024 Mar 6;19(3):e0298048. doi: 10.1371/journal.pone.0298048 (PMC10917258; doi:10.1371/journal.pone.0298048)
Supplement: S1 Table — (PDF) [file pone.0298048.s001.pdf]

S1 Table. Multiple logistic regression analysis (male)

Variables	Estimated coefficients	Standard error	z-value	OR	95% CI	*P*-value
Age (years)	0.193	0.015	12.718	1.212	1.178–1.250	<.001
BMI						
Normal				Reference		
Underweight	1.105	0.427	2.589	3.020	1.350–7.311	0.010
Obesity	-0.084	0.144	-0.582	0.920	0.694–1.218	0.561
Smoking						
Never				Reference		
Former	0.064	0.167	0.386	1.066	0.770–1.480	0.700
Current	0.669	0.192	3.480	1.953	1.342–2.854	0.001
Height (cm)	-0.024	0.012	-1.926	0.977	0.953–1.000	0.054

Nagelkerke R^2 =0.274.

Abbreviations: OR, odds ratio; CI, confidence interval; BMI, body mass index.
