# Supplementary material for: Sex differences in associated factors for age-related hearing loss
Source: PLoS One. 2024 Mar 6;19(3):e0298048. doi: 10.1371/journal.pone.0298048 (PMC10917258; doi:10.1371/journal.pone.0298048)
Supplement: S2 Table — (PDF) [file pone.0298048.s002.pdf]

S2 Table. Multiple logistic regression analysis (female)

Variables	Estimated coefficients	Standard error	z-value	OR	95% CI	*P*-value
Age (years)	0.183	0.015	12.526	1.200	1.167–1.236	<.001
Hypertension	0.212	0.158	1.344	1.237	0.906–1.684	0.179
Diabetes	0.313	0.237	1.319	1.367	0.854–2.166	0.187
BMI						
Normal				Reference		
Underweight	-0.005	0.439	-0.012	0.995	0.412–2.309	0.990
Obesity	0.743	0.245	3.034	2.102	1.315–3.434	0.002
Weight (kg)	-0.048	0.017	-2.891	0.953	0.921–0.983	0.004
Height (cm)	0.016	0.016	0.979	1.016	0.990–1.051	0.328
Age at menarche (years)	0.113	0.041	2.752	1.119	1.033–1.213	0.006

Nagelkerke R^2 =0.304.

Abbreviations: OR, odds ratio; CI, confidence interval; BMI, body mass index.
