# Supplementary material for: Sex differences in associated factors for age-related hearing loss
Source: PLoS One. 2024 Mar 6;19(3):e0298048. doi: 10.1371/journal.pone.0298048 (PMC10917258; doi:10.1371/journal.pone.0298048)
Supplement: Attachment — Submitted filename: Response_letter_231025_mhp.docx [file pone.0298048.s003.docx]

To Emily Chenette

Editor-in-Chief of *PLOS ONE*

Dear editor,

I, on behalf of all the authors, would like to thank you and the reviewers of *PLOS ONE* for taking the time and effort to review our manuscript, entitled “Sex differences in risk factors for age-related hearing loss” (Submission ID: PONE-D-23-24622). Your editorial staffs have provided us with a comprehensive review. Many of the valuable and constructive points that the reviewers pointed out were well taken by all the authors. After going over the reviewers’ comments, my colleagues and I made revisions and indicated the corrections and changes in yellow in the manuscript in the hope of improving our paper.

We hope that our revisions meet the editor’s and reviewers’ expectations. We believe that the comments have significantly improved the quality of our manuscript and hope you will find our revised manuscript acceptable for publication.

Specific answers to reviewer's comments start from page 2.

Sincerely yours,

Ji Bong Jeong, MD, PhD,

Division of Gastroenterology and Hepatology, Department of Internal Medicine, Bormae Medical Center, Seoul National University College of Medicine, 20 Borame-ro 5-gil, Dongjak-gu, Seoul 07061, South Korea

E-mail: jibjeong@snu.ac.kr

**Response to comment from the Reviewer #1:**

Comment 1:

Risk factors can’t be estimated from cross-sectional studies. This does not mean that this work is not a contribution to the field, but that authors should state that they are estimating associated factors, and discussing the potential bias derived from using this approach. It is critical to realize that odds ratio can vary substantially using cross-sectional vs longitudinal approaches.

**Response:** We followed your advice and changed all of the “risk factor” to “associated factor” in the body, including the title.

Sex differences in associated factors for age-related hearing loss

Comment 2: Variable definitions are not supported in previous literature. Also, risk factors are categorical variables, but many of the predictors were entered as continues variables and no cut-off point was estimated. This is again aligned with an associated factor rationale rather than a risk factor.

**Response:** We appreciate your feedback. We have provided references from existing literature to define age-related hearing loss, asymmetric hearing loss and obesity. And to redefine certain variables, specifically sarcopenia and visceral adiposity, we have employed cutoff values after consulting relevant literature. The script now reads (please refer to line 110-123):

ARHL was defined as a 2 kHz threshold greater than 25 dB in the better ear and a 4 kHz threshold equal to or greater than the 2 kHz threshold in the same ear, which represents a modification of the criteria originally proposed by Sousa, C. S. et al[18]. Hearing loss that did not fall into either of these categories was excluded from the analysis. Asymmetric hearing loss was defined as a difference of 20 dB or more in the average hearing threshold between the two ears and was excluded from the analysis[19]. Weight was measured in kg, and height was measured in cm. Obesity, underweight, visceral adiposity, and sarcopenia were used as the body composition parameters. Obesity was defined as BMI ≥ 25, while underweight was defined as BMI < 18.5[20]. Visceral adiposity was evaluated via VFA, measured using an InBody 720. Subjects whose VFA ≥ 100 ㎠ were categorized as part of the visceral adiposity group, while those with VFA < 100 ㎠ were assigned to the normal group[21]. ASM was calculated as the sum of the lean skeletal muscle masses of the bilateral upper and lower limbs. Sarcopenia was evaluated using ASM% calculated as ASM × 100/weight. Sarcopenia was defined as having an ASM% below 29.0 in males and under 22.9 in females[22].

Comment 3: The variables selected as potential risk factors should be properly addressed in the introduction. It is critical in this kind of study to present evidence supporting the link between the risk and the outcome to avoid spurious associations.

**Response:** We appreciate your suggestions, and as per your recommendations, we have improved the introduction and included references to the associated factors discussed in the main text. The script now reads (please refer to line 58-76):

Chronic noise exposure and ototoxic drugs are well-known risk factors for hearing loss. Strong associations have been reported between a family history of hearing impairment and ARHL[8]. ARHL has been reported to be associated with Caucasian ethnicity[9] and lower socioeconomic status[10]. Associations between hearing loss and smoking[11], alcohol[12], and other chronic diseases, such as hypertension[13], diabetes[14], dyslipidemia[15], obesity[16], underweight[17], and sarcopenia[18] have been reported. A higher risk of hearing loss has been reported for short stature[19] and adiposity[20].

In addition, there are differences in hearing loss according to sex: the prevalence is lower in females than in males[21] and the age at onset of hearing loss is later[22]. Research indicates a sex-specific association between factors related to abdominal fat and hearing loss, with males showing a correlation with high-frequency hearing loss, and females exhibiting a correlation with low-frequency hearing loss[23]. Research on risk factors for hearing loss has also noted sex-based distinctions, where, for instance, high triglyceride levels and a history of smoking have been identified as risk factors among males, while a high body mass index (BMI) has been associated with females[24].

In females, reproductive factors have been associated with various chronic conditions. For instance, associations have been reported between increased parity and breast cancer[25], age at menarche and cardiovascular diseases[26], as well as the duration of estrogen exposure and cardiovascular diseases[27]. However, research on the association between obstetric history and ARHL is currently limited.

Comment 4: Similarly, variables such as menopause are included, without specifying how is entered (I assume is present, absent, however is not declared). This variable is an event, not an exposition. As such, is difficult to follow the rationale of considering it a risk factor. This rationale, as those in the other proposed variables, must be explicit and supported in previous literature.

**Response:** We agree with your suggestion and have excluded menopause as an explanatory variable in subsequent analyses.

Comment 5: It was not entirely clear the context at which patient arrive to consultation. Are they preventive, routine, or other? This may contribute to potential bias not discussed in the discussion.

**Response:** We appreciate your feedback. The participants voluntarily sought healthcare services with a focus on health management and preventive purposes. This explanation has been added, and the corresponding limitations have been incorporated into the main text. The script now reads (please refer to line 83-85):

A total of 22,332 individuals who underwent medical examinations, including hearing tests, were screened. The context of their visit was primarily oriented toward healthcare and preventive healthcare objectives.

And the script now reads (please refer to line 290-294):

Furthermore, the presence of unilateral conductive or unilateral sudden hearing loss did not impact the results, as the analysis focused on better ear hearing and excluded cases of asymmetric hearing loss. In addition, this study was conducted among individuals who voluntarily visited health check-up centers for the purposes of health management and prevention. As a result, the study may not be representative of the general population, and, consequently, there exists the potential for bias.

Comment 6: Given the sex perspective of the study, a similar table to those reported in Table 1 should address descriptives by sex. For instance, many comments are given in the discussion of significant differences found in variables of Table 1, however, those differences are not divided by sex as logistic regressions.

**Response:** We appreciate your feedback. Simple logistic regression analyses were performed for each sex and are presented in Table 2.

**Table 2. Univariate Logistic Regression Analysis of the Association between Age-Related Hearing Loss and Multiple Explanatory Variables, Stratified by Sex**

| Variables | Male |  |  | Female |  |  |
| --- | --- | --- | --- | --- | --- | --- |
|  | OR | 95% CI | *P*-value | OR | 95% CI | *P*-value |
| Age | 1.209 | 1.175–1.245 | <.001 | 1.218 | 1.187–1.252 | <.001 |
| Hypertension | 1.119 | 0.881–1.422 | .358 | 1.745 | 1.355–2.251 | <.001 |
| Diabetes | 1.158 | 0.874–1.534 | .307 | 1.639 | 1.178–2.266 | .003 |
| Dyslipidemia | 1.016 | 0.800–1.289 | .897 | 1.465 | 1.135–1.894 | .003 |
| BMI |  |  |  |  |  |  |
| Normal | Reference |  |  | Reference |  |  |
| Obesity | 0.826 | 0.642–1.061 | .135 | 1.524 | 1.168–1.987 | .002 |
| Underweight | 3.557 | 1.710–8.119 | .001 | 1.806 | 0.919–3.421 | .076 |
| Visceral adiposity | 0.871 | 0.687–1.105 | .255 | 1.241 | 0.959–1.604 | .099 |
| Sarcopenia | 1.225 | 0.872–1.720 | .240 | 2.009 | 1.419–2.829 | <.001 |
| Alcohol | 0.684 | 0.476–0.980 | .039 | 0.809 | 0.623–1.054 | .115 |
| Smoking |  |  |  |  |  |  |
| Never | Reference |  |  | Reference |  |  |
| Former | 0.844 | 0.635–1.121 | .240 | 0.864 | 0.312–2.065 | .766 |
| Current | 1.241 | 0.894–1.726 | .198 | 0.821 | 0.183–2.703 | .758 |
| Weight (kg) | 0.973 | 0.960–0.985 | <.001 | 0.984 | 0.968–0.999 | .034 |
| Height (cm) | 0.956 | 0.935–0.976 | <.001 | 0.946 | 0.925–0.968 | <.001 |
| History of childbirth |  |  |  | 1.019 | 0.612–1.765 | .943 |
| Age at menarche (years) |  |  |  | 1.251 | 1.166–1.344 | <.001 |
| Hormone exposure duration (years) |  |  |  | 0.953 | 0.931–0.975 | <.001 |
| HRT |  |  |  | 0.949 | 0.660–1.345 | .773 |

Abbreviations: OR, odds ratio; CI, confidence interval; BMI, body mass index; HRT, hormone replacement therapy.

Comment 7: The usual approach when estimating ORs in associated factors is to run simple logistic regression, not multivariate. The reason to do so is that risk factors are not independent, and this affects the estimation of ORs. For the case of this multivariate model, authors not only include a high number of predictors, which also negatively affects GLMs, but they also don’t check for collinearity. Authors should check variance inflation factor if this approach is used. Many variables are most likely highly correlated, and given the number of regressors, collinearity is most likely high.

**Response:** We appreciate your feedback. It is not sufficient to estimate and interpret odds ratios from univariate analysis in the absence of controls. This is especially true when the relationship between an outcome and a risk factor is confounded by confounders that are not adequately controlled[1]. In such cases, the impact of the risk factor in question may be inaccurately assessed. This is why we conducted multiple logistic regression analyses in this study.

For example, smoking was reported as a significant risk factor for males in the existing literature[2]. However, in this study, it was not significant in univariate analysis. Nevertheless, after selecting variables and adjusting for age, sex, and BMI, smoking was found to have a statistically significant relationship with age-related hearing loss.

Furthermore, when we examined multicollinearity, the Variance Inflation Factor (VIF) values were no higher than 10 in both the full model, which included all potential variables, and the simplified model obtained through variable selection.

We apologize for not fully addressing these points in the original manuscript and have added them to the text as you pointed out. The script now reads (please refer to line 230-232):

Smoking, initially non-significant in univariate analysis, later proved significant in variable selection, highlighting the risk of excluding important variables when using univariate regression, consistent with prior research[36].

And the script now reads (please refer to line 264-269):

As exemplified in the case of age and sarcopenia mentioned above, when introducing multiple explanatory variables in multiple regression, it is imperative to consider multicollinearity. In this study, variance inflation factor (VIF) was computed for both the multiple regression model utilizing all variables and the simplified model obtained after variable selection. For all coefficients in both models, VIF values did not exceed 10.

Comment 8: Finally, considering the sample size, authors present high sensibility to detect minor but not relevant differences. This is not discussed. Only p-values are considered, without neglecting differences that may be considered as fairly irrelevant. Also, ORs estimated cross-sectionally, tend to present higher ORs. Considering these two elements, only a few results from the entire outcome would be promising for future longitudinal studies.

**Response:** We concur with your feedback. This study is cross-sectional in nature, which may have led to relatively elevated odds ratio values. Furthermore, due to the limited sample size in this study, some of the variables that were found significant here may only be significant in certain large-scale studies. Therefore, when interpreting the results of this study, it is essential to consider these limitations. The script now reads (please refer to line 279-284):

As this is a cross-sectional study, it is not possible to derive a causal relationship; therefore, further studies are required to confirm these findings. Additionally, the cross-sectional design of this study could have led to relatively elevated odds ratio values. Furthermore, owing to the limited sample size in this study, it's possible that only a subset of the variables considered significant here might remain significant in a larger study. Consequently, these limitations should be taken into account when interpreting the study's results.

**Response to comment from the Reviewer #2:**

Comment 1: The main objective of the paper should be assessment of sex differences in risk factors for age related hearing loss (ARHL), since this is the tittle. Nevertheless, in the introduction there is only one phrase discussing this topic from previous publications. The authors should expand on this aspect so the reader can clearly understand the objective of the paper, or change the tittle.

**Response:** We appreciate your suggestions and have enhanced the introduction, incorporating additional literature to address sex differences in risk factors for hearing loss as per your recommendations. The script now reads (please refer to line 65-76):

In addition, there are differences in hearing loss according to sex: the prevalence is lower in females than in males[21] and the age at onset of hearing loss is later[22]. Research indicates a sex-specific association between factors related to abdominal fat and hearing loss, with males showing a correlation with high-frequency hearing loss, and females exhibiting a correlation with low-frequency hearing loss[23]. Research on risk factors for hearing loss has also noted sex-based distinctions, where, for instance, high triglyceride levels and a history of smoking have been identified as risk factors among males, while a high body mass index (BMI) has been associated with females[24].

In females, reproductive factors have been associated with various chronic conditions. For instance, associations have been reported between increased parity and breast cancer[25], age at menarche and cardiovascular diseases[26], as well as the duration of estrogen exposure and cardiovascular diseases[27]. However, research on the association between obstetric history and ARHL is currently limited.

Comment 2: The diagnosis of ARHL in the clinical setting usually relies on symmetric descending sensorineural hearing loss. Even though there is no clear age limit for the onset of this condition, most of the literature on the topic include adults over the age of 60 years old. In this paper, the age range spans between 40 and 94 years of age. Because of this, genetic-related adult-onset hearing loss could be included along with ARHL. My recommendation for the authors is to analyze the data again including only subjects older than 60 years of age.

**Response:** We appreciate your feedback. We ran a new analysis restricting subjects to those aged 60 and older. The script now reads (please refer to line 86-88):

Among them, 2,349 participants were analyzed, excluding those younger than 60, those with missing data, those with a history of occupational noise exposure, those with hearing loss other than ARHL, those with asymmetric hearing loss, and those with central nervous system diseases (Fig 1).

Comment 3: Also, there is no mention of asymmetric hearing loss. This is not typical of ARHL and these cases should be excluded.

**Response:** We appreciate your feedback. In this study, we referred to the criterion for asymmetric hearing loss proposed by Kurioka et al., which is "AC threshold between the right and left ear (RL-gap) >= 20 dB."[3] An explanation of this criterion has also been included in the main text. The script now reads (please refer to line 86-88):

Among them, 2,349 participants were analyzed, excluding those younger than 60, those with missing data, those with a history of occupational noise exposure, those with hearing loss other than ARHL, those with asymmetric hearing loss, and those with central nervous system diseases (Fig 1).

And the script now reads (please refer to line 114-115):

Asymmetric hearing loss was defined as a difference of 20 dB or more in the average hearing threshold between the two ears and was excluded from the analysis[30].

Comment 4: There are some known risk factors for hearing loss in adults, such as head trauma or infectious diseases, that are not included. Please explain why.

**Response:** We appreciate your feedback. This study utilized information from questionnaire items conducted during health check-ups, and we could not investigate the past history of head injuries or infectious diseases because these were not included in the questionnaire items. This constitutes a limitation of our study, and we have added a mention of this limitation. The script now reads (please refer to line 284-286):

At the time of data collection, the associated factors related to hearing loss, such as history of ototoxic drug exposure, family history of hearing loss, history of head trauma and infectious diseases, were not assessed.

Comment 5: Regarding hormonal exposure, there is no mention of menopausal hormonal replacement therapy. If there is no data this issue should be included in the discussion.

**Response:** We appreciate your feedback. The use of hormone replacement therapy in postmenopausal participants was investigated, and this has been included in the main text. However, the duration of hormone replacement therapy was not examined, which represents a limitation of the present study. The script now reads (please refer to line 273-278):

However, it's notable that while age at menarche was included as a variable, the duration of hormone exposure was excluded from the model. In our study, the duration of hormone exposure was defined as the timespan from menarche to menopause, irrespective of HRT utilization or its duration. In this study, we investigated the use of HRT, but we did not assess the duration of treatment, resulting in an incomplete analysis of its potential effects. Therefore, further comprehensive research is imperative to address this aspect comprehensively.

Comment 6: Table 1 should include intervals of confidence. Also I would recommend to change the % to make reference to the total according to hearing status and not to the total of subjects. For example, in sex distribution “Male” would be 51.1% in normal hearing and 64.1% in ARHL.

**Response:** We appreciate your feedback. To handle confidence intervals, we conducted univariate logistic regression analysis with stratification by sex, and the results have been summarized in Table 2. As you pointed out, we have made the necessary revisions to the notation in Table 1.

**Table 1. Participants characteristics**

| Variables | Total  n=2,349 | Normal hearing  n=1,520 | ARHL  n=829 | *P*-value |
| --- | --- | --- | --- | --- |
| Age | 66.5±5.6 | 64.7±4.2 | 69.8±6.3 | <.001 |
| Sex |  |  |  | <.001 |
| Male | 1,104 (47) | 607 (40) | 497 (60) |  |
| Female | 1,245 (53) | 913 (60) | 332 (40) |  |
| Hypertension | 1,198 (51) | 723 (48) | 475 (57) | <.001 |
| Diabetes | 445 (19) | 256 (17) | 189 (23) | <.001 |
| Dyslipidemia | 1,165 (50) | 740 (49) | 425 (51) | .249 |
| BMI |  |  |  | <.001 |
| Normal | 1,470 (63) | 975 (64) | 495 (60) |  |
| Obesity | 801 (34) | 509 (34) | 292 (35) |  |
| Underweight | 78 (3.3) | 36 (2.4) | 42 (5.1) |  |
| Visceral adiposity | 1001 (43) | 633 (42) | 368 (44) | .214 |
| Sarcopenia | 317 (14) | 176 (12) | 141 (17) | <.001 |
| Alcohol | 1,800 (77) | 1,165 (77) | 635 (77) | 1.000 |
| Smoking |  |  |  | <.001 |
| Never | 1,511 (64) | 1,049 (69) | 462 (56) |  |
| Former | 545 (23) | 321 (21) | 224 (27) |  |
| Current | 293 (13) | 150 (9.9) | 143 (17) |  |
| Weight (kg) | 61.6±10.4 | 61.5±10.2 | 61.7±10.6 | .611 |
| Height (cm) | 160.6±9.1 | 160.3±9.2 | 161.2±8.8 | .021 |

Abbreviations: ARHL, age-related hearing loss; BMI, body mass index

# References

1.	Sun GW, Shook TL, Kay GL. Inappropriate use of bivariable analysis to screen risk factors for use in multivariable analysis. J Clin Epidemiol. 1996;49(8):907-16.

2.	Kumar A, Gulati R, Singhal S, Hasan A, Khan A. The effect of smoking on the hearing status-a hospital based study. J Clin Diagn Res. 2013;7(2):210-4.

3.	Kurioka T, Sano H, Furuki S, Yamashita T. Speech discrimination impairment of the worse-hearing ear in asymmetric hearing loss. Int J Audiol. 2021;60(1):54-9.
