# Supplementary material for: Sex differences in associated factors for age-related hearing loss
Source: PLoS One. 2024 Mar 6;19(3):e0298048. doi: 10.1371/journal.pone.0298048 (PMC10917258; doi:10.1371/journal.pone.0298048)
Supplement: Attachment — Submitted filename: Response_letter_240110.docx [file pone.0298048.s004.docx]

Paul Hinckley Delano, Ph.D.

Editor-in-Chief of *PLOS ONE*

Dear editor,

I, on behalf of all the authors, would like to thank you and the reviewers of *PLOS ONE* for taking the time and effort to review our manuscript, entitled “Sex differences in associated factors for age-related hearing loss” (Submission ID: PONE-D-23-24622). Your editorial staffs have provided us with a comprehensive review. Many of the valuable and constructive points that the reviewers pointed out were well taken by all the authors. After going over the reviewers’ comments, my colleagues and I made revisions and indicated the corrections and changes in yellow in the manuscript in the hope of improving our paper.

We hope that our revisions meet the editor’s and reviewers’ expectations. We believe that the comments have significantly improved the quality of our manuscript and hope you will find our revised manuscript acceptable for publication.

Specific answers to reviewer's comments start from page 2.

Sincerely yours,

Ji Bong Jeong, MD, PhD,

Division of Gastroenterology and Hepatology, Department of Internal Medicine, Bormae Medical Center, Seoul National University College of Medicine, 20 Borame-ro 5-gil, Dongjak-gu, Seoul 07061, South Korea

E-mail: jibjeong@snu.ac.kr

**Response to comment from the Reviewer #1:**

Comment 1:

From line 115 to 125 seems to be a change in format.

In line 142 an space is missing after "elimination"

**Response:** We appreciate your feedback. The formatting issue you highlighted has been addressed. The script now reads (please refer to line 117-127):

Obesity was defined as BMI $\geq$ 25, while underweight was defined as BMI $<$ 18.5. Visceral adiposity was evaluated via VFA, measured using an InBody 720. Subjects whose VFA $\geq$ 100 ${cm}^{2}$ were categorized as part of the visceral adiposity group, while those with VFA $<$ 100 ${cm}^{2}$ were assigned to the normal group. ASM was calculated as the sum of the lean skeletal muscle masses of the bilateral upper and lower limbs. Sarcopenia was evaluated using ASM% calculated as ASM $\times$ 100/weight. Sarcopenia was defined as having an ASM% below 29.0 in males and under 22.9 in females. Hypertension was defined as systolic blood pressure $\geq$ 140 mmHg, diastolic blood pressure $\geq$ 90 mmHg, or the use of antihypertensive medications. Diabetes was defined as fasting glucose $\geq$ 126 mg/dL, glycated hemoglobin level $\geq$ 6.5%, or use of antidiabetic medications. Dyslipidemia was defined as triglyceride level $\geq$ 150 mg/dL, HDL-C $<$ 40 mg/dL in males and $<$ 50 mg/dL in females, or the use of anti-dyslipidemia medications.

And we have also added the missing spaces as indicated. The script now reads (please refer to line 143):

Variable selection was performed using the augmented backward elimination (ABE) method,

Comment 2:

I understand the use of BMI, but not so clear heigh. Even weight alone is easy to infer why is entered in the model. But heigh alone does not make much sense, and there is no mention to it in the introduction explaining a potential causal role in audition loss. I suggest the authors include information in the introduction regarding height or removing it.

**Response:** We sincerely appreciate your valuable feedback. We acknowledge that our initial manuscript did not adequately address the relationship between height and hearing loss. In response to your suggestion, we have revised the Introduction section to include a reference to previous study that explore this relationship. Additionally, we have elaborated on the authors' hypothesis concerning this correlation. We believe these amendments will significantly strengthen our manuscript and thank you for your insightful suggestions. The script now reads (please refer to line 61-64):

Associations between hearing loss and smoking[11], alcohol[12], and other chronic diseases, such as hypertension[13], diabetes[14], dyslipidemia[15], obesity[16], underweight[17], sarcopenia[18] and adiposity[19] have been reported. Short stature has been reported to be associated with a higher risk of hearing loss, potentially due to fetal growth issues involving insulin-like growth factor 1, which affects cochlear development[20].

Comment 3:

Finally, please provide a reference for VIF cut off =10. That is a rather high cut off value (removing high should reduce VIF values anyway). Also, this information should be provided in methods rather than discussion.

**Response:** We are grateful for your insightful feedback. We concur with your observation that a threshold of 10 for the Variance Inflation Factor (VIF) is excessively high to effectively rule out multicollinearity. Upon re-evaluating our models, including the variable 'height,' we found that the highest VIF value recorded is 4.6 for 'weight.' This value is well within the widely accepted threshold of 5 for VIF values, indicating a lower risk of multicollinearity.

Furthermore, we agree with your recommendation that this analysis should be included in the Methods section rather than the Discussion. We have revised our manuscript to reflect this change, ensuring that our approach to addressing multicollinearity is clearly outlined in the appropriate section. Thank you for guiding these improvements in our manuscript. The script now reads (please refer to line 147-152):

To assess multicollinearity in our regression models, we calculated the variance inflation factor (VIF). Initially, the VIF was computed for the multiple regression model incorporating all the considered variables. This was followed by computing the VIF for the simplified model, which was derived after the process of variable selection. The purpose of this analysis was to ensure that multicollinearity remains within acceptable limits, defined by having all coefficients in both models below the threshold value of 5.

**Response to comment from the Reviewer #3:**

I only have reservations about the conclusion which, in general, is very similar to the three previous lines before the discussion section (I suggest deleting lines 294 to 296 from the paper)

**Response:** We appreciate your feedback. We've revised the manuscript as you pointed out.
